# Prevalence of Disability and Disability Type Among Adults — United States, 2013

**DOI:** 10.15585/mmwr.mm6429a2

**Published:** 2015-07-31

**Authors:** Elizabeth A. Courtney-Long, Dianna D. Carroll, Qing C. Zhang, Alissa C. Stevens, Shannon Griffin-Blake, Brian S. Armour, Vincent A. Campbell

**Affiliations:** 1 Division of Human Development and Disability, National Center on Birth Defects and Developmental Disabilities, CDC; 2 Commissioned Corps, U.S. Public Health Service, CDC

Understanding the prevalence of disability is important for public health programs to be able to address the needs of persons with disabilities. Beginning in 2013, to measure disability prevalence by functional type, the Behavioral Risk Factor Surveillance System (BRFSS), added five questions* to identify disability in vision, cognition, mobility, self-care, and independent living.† CDC analyzed data from the 2013 BRFSS to assess overall prevalence of any disability, as well as specific types of disability among noninstitutionalized U.S. adults. Across all states, disabilities in mobility and cognition were the most frequently reported types. State-level prevalence of each disability type ranged from 2.7% to 8.1% (vision); 6.9% to 16.8% (cognition); 8.5% to 20.7% (mobility); 1.9% to 6.2% (self-care) and 4.2% to 10.8% (independent living). A higher prevalence of any disability was generally seen among adults living in states in the South and among women (24.4%) compared with men (19.8%). Prevalences of any disability and disability in mobility were higher among older age groups. These are the first data on functional disability types available in a state-based health survey. This information can help public health programs identify the prevalence of and demographic characteristics associated with different disability types among U.S. adults and better target appropriate interventions to reduce health disparities.

BRFSS is an annual state-based random-digit–dialed telephone (landline and cell phone) survey of the U.S. noninstitutionalized civilian population aged ≥18 years. During 2013, the median response rate among the 50 states and District of Columbia (DC) was 45.9% and ranged from 29.0% (Alabama) to 59.2% (North Dakota).§ The 2013 survey included, for the first time, questions about five disability types (vision, cognition, mobility, self-care, and independent living).¶ Respondents were identified as having one of the five disability types if they answered “yes” to the relevant question. Respondents who responded “yes” to at least one of the disability questions were identified as having any disability. Responses of “don’t know” or “refused” were excluded from analyses. Prevalences of any disability and disability type (with 95% confidence intervals) were calculated by state, sex, age group, race/ethnicity, veteran status, annual household income, employment status, and education level. All estimates were age-adjusted to the 2000 U.S. population. Data were weighted and analyzed to account for the complex sampling design of BRFSS. Two-sided chi-square tests were used to compare prevalence estimates between demographic subgroups.

Overall, 22.2% of U.S. adults (53,316,677 persons) reported any disability. Disability in mobility was the most frequently reported type (13.0%), followed by disability in cognition (10.6%), independent living (6.5%), vision (4.6%), and self-care (3.6%) (Table 1).

Prevalence of any disability differed across states, ranging from 16.4% (Minnesota) to 31.5% (Alabama). Prevalences of each disability type also varied across states. Disability in vision ranged from 2.7% in Idaho and New Hampshire to 8.1% in Mississippi; disability in cognition ranged from 6.9% in North Dakota and South Dakota to 16.8% in Arkansas; disability in mobility ranged from 8.5% in Minnesota to 20.7% in Mississippi; self-care disability ranged from 1.9% in Hawaii to 6.2% in Mississippi; and disability in independent living ranged from 4.2% in Nebraska and Utah to 10.8% in Mississippi. Generally, states with higher disability prevalences were located in the South and those with lower prevalences were in the Midwest or West (Table 1; Figure).

Women reported a higher prevalence of any disability (24.4%) than did men (19.8%), and also reported higher prevalences of each disability type. Prevalences of any disability and of each type were highest in either the oldest age group (≥65 years) or both the middle (45–64 years) and oldest age groups, with the exception of cognition, where the reported prevalence was highest among persons aged 45–64 years (12.0% versus 10.1% [18–44 years] and 9.9% [≥65 years]). Black, non-Hispanic adults reported the highest prevalences of any disability and of each disability type; the highest prevalence of disability in vision (7.4%) was the same among black, non-Hispanic adults and Hispanic adults. Compared with veterans, non-veterans reported a higher prevalence of disability in vision (4.7% versus 3.9%) and independent living (6.7% versus 5.9%). Respondents with higher household income levels and higher levels of education had lower prevalences of any disability and of each disability type. Nearly 50% of adults with a household income of <$15,000 and 40% of adults who did not graduate from high school had any disability compared with only 10.8% of adults with a household income of ≥$50,000 and 11.8% of college graduates, respectively. Prevalence of any disability among unemployed adults was more than twice as high as it was among those who were employed. (33.5% versus 12.6%) (Table 2).

## Discussion

In 2000, in the first report of state-based data on disability that was generated from the 1998 BRFSS data (1),** CDC described a median state-level disability prevalence of 17.1% in 11 states and DC. Since 2003, BRFSS has assessed disability†† in all participating states and territories using two questions regarding activity limitation and special equipment use. These questions, however, did not address the type of functional limitation or condition associated with the disability. In 2011, pursuant to Section 4302 of the Affordable Care Act,§§ the Department of Health and Human Services issued guidance for defining and collecting data on disability status using a standard set of questions. On the basis of this guidance,† BRFSS added five additional disability questions to the survey in 2013.*

In 2013, approximately one in five U.S. adults reported any disability, with state-level prevalence of any disability ranging from 16.4% in Minnesota to 31.5% in Alabama. Reasons behind state-level differences in disability are unclear; however, disability prevalence was generally higher in the South, a region noted to have one of the higher prevalences of social determinants of poor health (2,3), which are also associated with disability (2–4). The higher overall prevalence of disability in this report compared with the 2000 report might be explained, in part, by the use of different operational definitions of disability, a true increase in prevalence in the 15 years since the 1998 survey, or the inclusion of all states and DC in this report.

Many findings in this study are consistent with earlier reports. Previous research found lower education levels among adults with a disability compared with those without (5); in this study, approximately 40% of those who did not complete high school reported any disability. Public health programs for persons with one or more disabilities might need to account for this, as lower health literacy has been associated with lower education levels (6). The most frequently reported disability type was mobility, which is consistent with other findings (7): the top two causes of disability are associated with physical or mobility limitations (arthritis, back and spine problems) and account for over 35% of all disability (8).

Disability prevalence has been shown to increase with age (5). Although the prevalences of any disability and disabilities in mobility and independent living increased with age, this was not the case for disabilities in vision, cognition and self-care. This observed association of any disability with increasing age might be because of disability in mobility; at 13%, this was the most frequently reported disability type, and disability in mobility increases with age. Prevalences of vision and self-care disability were similar for adults aged 45–64 years and adults aged ≥65 years. In contrast, the highest prevalence of disability in cognition was among adults aged 45–64 years. This could be accounted for, in part, by the exclusion from the survey of adults living in institutional settings, as older adults may be more likely to live in such settings (e.g., nursing homes) than younger adults. In addition, although underlying medical conditions are not ascertained in BRFSS, many middle-aged adults who indicated a limitation in cognition during development and testing of that question also reported having mental illness (9). Furthermore, among all disability types, the largest increases in prevalence occurred between persons aged 18–44 years and those aged 45–64 years (e.g., the prevalence of mobility disability was more than three times higher among persons aged 45–64 years compared with those aged 18–44 years). Understanding the age profiles of different disability types can enhance the development of age- and disability-inclusive public health programs.

**Summary**

What is already known on this topic?

Disability has been measured in numerous ways in national health surveys. CDC previously used 1998 BRFSS data to report disability prevalence in 11 states and the District of Columbia (DC). The median disability prevalence, using a nonspecific definition of disability, was 17.1%.

What is added by this report?

Five questions added to the 2013 BRFSS were used to measure functional disability type in the 50 states and DC. Overall prevalence of any disability was 22.2%; the most frequently reported disability types were mobility (mean = 13.0%) and cognition (mean = 10.6%). In general, disability prevalences were higher among women, adults ≥65 years of age, racial/ethnic minorities, persons with annual household incomes <$15,000 per year, and those who had less than a high school education.

What are the implications for public health practice?

More than 53 million U.S. adults reported a disability in 2013. Since disability among adults is associated with disparities in behavioral risk factors for health (e.g., smoking and physical inactivity), more specific information on disability and disability types will inform public health researchers and program planners to better understand the relationships between disability, demographic factors, and health status to identify and address barriers to more effective interventions.

The findings in this report are subject to at least four limitations. First, all BRFSS data are self-reported and, therefore, might be subject to recall and social desirability bias. However, self-reporting is the most commonly used method for assessing disability for surveillance purposes. Second, nonresponse bias is possible because response rates among the states and DC ranged from 29.0% to 59.2% (median: 45.9%). Third, because three of the disability questions include the modifier “serious,” they might not identify respondents with more moderate limitations or who do not perceive their disabilities to be serious. Finally, BRFSS does not include adults living in institutional settings or group homes, which might systematically exclude persons with disabilities, since persons residing in these settings might be more likely to have a disability. Because these last two limitations can result in an underestimation of the disability prevalence and profile among all U.S. adults, the estimates reported here are likely to be conservative.

Disability has been associated with health disparities in behavioral risk factors (e.g., smoking and physical inactivity) and preventive health measures (e.g., mammography) (4). Maintaining health among people with disabilities is important, as annual disability-associated health care expenditures were estimated at nearly $400 billion in 2006, with over half attributable to costs related to non-independent living (e.g., institutional care, personal care services) (10). The ability of state programs to address these and other important public health needs among adults with disabilities has possibly been hindered by a lack of information on specific disability types. Having information about disability types, the demographic profiles of persons with different disability types, and health disparities associated with disabilities¶¶ will better enable researchers and program planners to make more focused, data-driven decisions and modify existing interventions to more effectively improve the health of persons with disabilities.

## Figures and Tables

**FIGURE:**
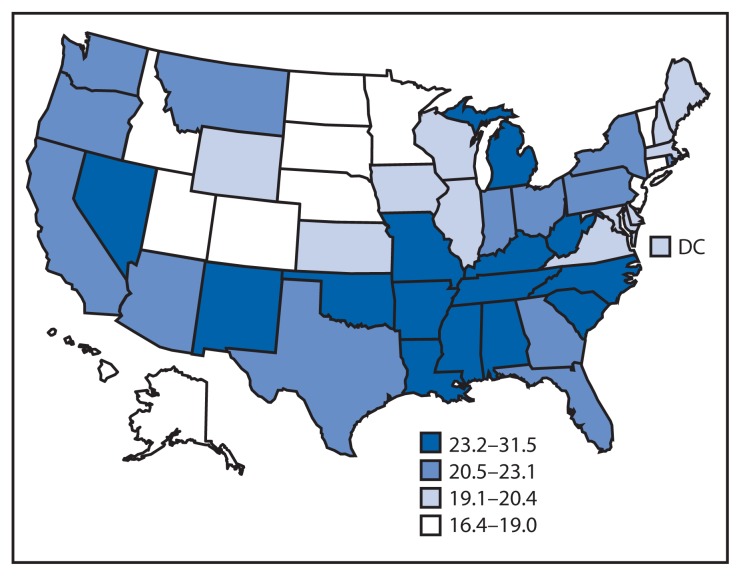
Prevalence* of any disability among adults aged ≥18 years — Behavioral Risk Factor Surveillance System, United States, 2013 Abbreviation: DC = District of Columbia. * Weighted estimates, age-adjusted to the 2000 U.S. standard population.

**TABLE 1:** Prevalence* of any disability and disability type,† by state,§ among adults aged ≥18 years — Behavioral Risk Factor Surveillance System, United States, 2013

State/Territory	Respondents** (No.)	Disability type¶											

		Vision		Cognition		Mobility		Self-care		Independent living		Any	
					
		(%)	(95% CI)	(%)	(95% CI)	(%)	(95% CI)	(%)	(95% CI)	(%)	(95% CI)	(%)	(95% CI)
**Overall**	**465,053**	**(4.6)**	**(4.5–4.7)**	**(10.6)**	**(10.4–10.8)**	**(13.0)**	**(12.8–13.2)**	**(3.6)**	**(3.5–3.7)**	**(6.5)**	**(6.4–6.7)**	**(22.2)**	**(21.9–22.4)**
Alabama	6,333	(7.5)	(6.6–8.5)	(16.3)	(15.0–17.8)	(20.6)	(19.3–21.9)	(5.3)	(4.6–6.0)	(10.1)	(9.2–11.1)	(31.5)	(29.9–33.2)
Alaska	4,429	(3.1)	(2.5–3.9)	(7.7)	(6.6–9.1)	(10.3)	(9.1–11.6)	(3.1)	(2.5–3.9)	(4.4)	(3.7–5.3)	(17.7)	(16.1–19.4)
Arizona	4,078	(4.8)	(3.5–6.5)	(10.9)	(9.3–12.7)	(12.4)	(10.8–14.2)	(3.7)	(2.6–5.2)	(6.9)	(5.5–8.6)	(21.8)	(19.6–24.2)
Arkansas	5,041	(7.2)	(6.1–8.4)	(16.8)	(15.2–18.5)	(18.0)	(16.6–19.5)	(5.3)	(4.4–6.3)	(9.1)	(8.0–10.2)	(30.0)	(28.1–31.9)
California	10,552	(5.4)	(4.8–6.0)	(9.8)	(9.0–10.6)	(11.4)	(10.7–12.3)	(3.4)	(3.0–3.9)	(5.6)	(5.0–6.2)	(20.9)	(19.9–22.0)
Colorado	12,687	(2.9)	(2.5–3.3)	(9.0)	(8.3–9.7)	(9.5)	(8.9–10.1)	(2.5)	(2.2–2.9)	(4.8)	(4.3–5.3)	(17.5)	(16.7–18.4)
Connecticut	7,411	(3.4)	(2.7–4.2)	(8.5)	(7.5–9.7)	(9.8)	(9.0–10.6)	(2.7)	(2.2–3.2)	(5.8)	(5.0–6.7)	(18.2)	(16.9–19.6)
Delaware	5,004	(4.1)	(3.5–4.9)	(9.9)	(8.7–11.2)	(12.1)	(11.0–13.2)	(3.1)	(2.5–3.9)	(5.9)	(5.1–6.9)	(20.2)	(18.7–21.8)
District of Columbia	4,683	(4.6)	(3.7–5.6)	(9.7)	(8.4–11.3)	(12.4)	(11.1–13.7)	(3.9)	(3.2–4.8)	(6.8)	(5.9–7.9)	(20.3)	(18.6–22.1)
Florida	32,652	(5.0)	(4.5–5.6)	(11.1)	(10.3–12.0)	(13.2)	(12.5–14.0)	(3.8)	(3.4–4.3)	(6.4)	(5.8–7.0)	(23.1)	(22.0–24.2)
Georgia	7,686	(5.0)	(4.4–5.7)	(10.6)	(9.7–11.6)	(14.2)	(13.3–15.1)	(3.5)	(3.0–4.1)	(6.2)	(5.6–7.0)	(23.0)	(21.8–24.3)
Hawaii	7,637	(3.6)	(3.0–4.3)	(7.6)	(6.7–8.5)	(9.1)	(8.3–10.0)	(1.9)	(1.6–2.4)	(5.1)	(4.4–5.9)	(17.5)	(16.3–18.7)
Idaho	5,440	(2.7)	(2.2–3.3)	(8.8)	(7.8–10.0)	(9.7)	(8.8–10.7)	(2.7)	(2.1–3.3)	(5.1)	(4.4–5.9)	(17.2)	(15.9–18.6)
Illinois	5,511	(3.9)	(3.1–4.8)	(8.3)	(7.2–9.5)	(10.9)	(9.8–11.9)	(2.8)	(2.2–3.5)	(5.5)	(4.7–6.4)	(19.1)	(17.6–20.7)
Indiana	9,966	(3.9)	(3.5–4.4)	(10.8)	(10.0–11.7)	(13.7)	(12.9–14.6)	(3.9)	(3.4–4.4)	(6.8)	(6.2–7.4)	(22.6)	(21.5–23.7)
Iowa	7,935	(3.4)	(2.9–3.9)	(9.3)	(8.3–10.4)	(11.0)	(10.2–11.8)	(2.9)	(2.5–3.4)	(5.1)	(4.4–5.8)	(19.2)	(18.0–20.4)
Kansas	22,781	(3.3)	(3.1–3.6)	(9.3)	(8.8–9.8)	(12.6)	(12.2–13.1)	(3.1)	(2.9–3.4)	(5.4)	(5.1–5.7)	(20.4)	(19.8–21.0)
Kentucky	10,536	(7.0)	(6.2–7.8)	(14.8)	(13.7–15.9)	(18.5)	(17.5–19.6)	(5.0)	(4.4–5.7)	(9.7)	(8.8–10.6)	(29.2)	(27.8–30.5)
Louisiana	5,128	(5.2)	(4.5–6.1)	(13.9)	(12.3–15.7)	(16.6)	(15.3–18.0)	(5.2)	(4.3–6.3)	(9.2)	(8.0–10.5)	(27.4)	(25.5–29.4)
Maine	7,911	(3.0)	(2.5–3.7)	(11.2)	(10.1–12.4)	(10.7)	(9.8–11.6)	(2.6)	(2.2–3.1)	(5.8)	(5.1–6.6)	(20.2)	(19.0–21.6)
Maryland	12,459	(3.2)	(2.8–3.8)	(9.3)	(8.4–10.3)	(11.7)	(11.0–12.6)	(3.0)	(2.5–3.5)	(5.6)	(4.9–6.3)	(19.9)	(18.8–21.1)
Massachusetts	14,200	(3.2)	(2.8–3.7)	(10.4)	(9.5–11.4)	(10.5)	(9.8–11.3)	(2.7)	(2.3–3.1)	(6.4)	(5.8–7.2)	(19.6)	(18.5–20.7)
Michigan	12,429	(4.7)	(4.1–5.2)	(12.0)	(11.1–12.9)	(14.8)	(14.0–15.7)	(4.4)	(3.9–5.0)	(7.5)	(6.8–8.2)	(24.6)	(23.5–25.7)
Minnesota	13,689	(2.8)	(2.3–3.4)	(7.7)	(6.9–8.6)	(8.5)	(7.7–9.3)	(2.1)	(1.7–2.5)	(4.4)	(3.8–5.1)	(16.4)	(15.2–17.5)
Mississippi	7,217	(8.1)	(7.3–9.0)	(15.7)	(14.4–17.0)	(20.7)	(19.5–21.9)	(6.2)	(5.4–7.0)	(10.8)	(9.7–11.9)	(31.4)	(29.8–32.9)
Missouri	6,940	(4.5)	(3.8–5.3)	(12.3)	(11.1–13.6)	(14.7)	(13.6–15.9)	(4.3)	(3.7–5.0)	(8.2)	(7.3–9.3)	(24.0)	(22.5–25.6)
Montana	9,485	(4.4)	(3.9–5.0)	(10.2)	(9.3–11.1)	(12.0)	(11.1–12.9)	(3.1)	(2.6–3.7)	(5.3)	(4.7–6.0)	(20.8)	(19.6–21.9)
Nebraska	16,605	(3.1)	(2.8–3.6)	(8.3)	(7.6–9.0)	(10.7)	(10.1–11.5)	(2.4)	(2.0–2.8)	(4.2)	(3.8–4.7)	(17.9)	(17.0–18.9)
Nevada	4,921	(4.5)	(3.6–5.6)	(12.0)	(10.2–14.0)	(13.2)	(11.8–14.8)	(3.1)	(2.4–4.0)	(6.1)	(5.1–7.4)	(23.7)	(21.5–26.0)
New Hampshire	6,214	(2.7)	(2.2–3.3)	(10.0)	(8.9–11.2)	(9.9)	(9.0–10.9)	(2.8)	(2.2–3.5)	(5.7)	(4.9–6.6)	(19.4)	(17.9–20.9)
New Jersey	12,486	(3.5)	(3.1–4.0)	(8.5)	(7.7–9.3)	(11.1)	(10.4–11.9)	(2.7)	(2.4–3.0)	(4.9)	(4.4–5.5)	(19.0)	(18.0–20.0)
New Mexico	9,025	(5.0)	(4.5–5.6)	(11.4)	(10.5–12.3)	(13.9)	(13.0–14.8)	(4.5)	(3.9–5.2)	(8.3)	(7.5–9.2)	(23.7)	(22.5–25.0)
New York	8,517	(4.5)	(4.0–5.2)	(10.1)	(9.2–11.1)	(12.9)	(12.1–13.8)	(3.5)	(3.0–4.0)	(6.7)	(6.0–7.4)	(22.1)	(20.9–23.3)
North Carolina	8,634	(4.8)	(4.3–5.5)	(11.5)	(10.6–12.5)	(14.9)	(14.0–15.8)	(4.3)	(3.8–4.9)	(7.5)	(6.8–8.3)	(24.3)	(23.1–25.5)
North Dakota	7,591	(2.9)	(2.4–3.6)	(6.9)	(6.0–7.8)	(10.0)	(9.2–10.9)	(2.3)	(1.9–2.9)	(4.4)	(3.7–5.2)	(16.5)	(15.3–17.7)
Ohio	11,417	(4.5)	(3.9–5.1)	(11.1)	(10.2–12.0)	(13.8)	(12.9–14.7)	(3.5)	(3.0–4.0)	(7.2)	(6.5–8.0)	(22.7)	(21.6–24.0)
Oklahoma	8,122	(6.2)	(5.5–7.0)	(14.5)	(13.5–15.7)	(17.5)	(16.5–18.4)	(4.7)	(4.2–5.2)	(8.6)	(7.8–9.4)	(28.2)	(26.9–29.5)
Oregon	5,741	(4.2)	(3.5–5.1)	(11.3)	(10.1–12.5)	(11.0)	(10.0–12.1)	(3.0)	(2.5–3.6)	(6.3)	(5.4–7.2)	(21.7)	(20.2–23.2)
Pennsylvania	11,022	(3.5)	(3.0–4.0)	(9.5)	(8.7–10.3)	(12.5)	(11.7–13.2)	(3.2)	(2.8–3.7)	(6.5)	(5.9–7.1)	(20.8)	(19.8–21.9)
Rhode Island	6,257	(4.1)	(3.4–4.8)	(12.1)	(10.8–13.5)	(12.6)	(11.6–13.6)	(3.1)	(2.6–3.7)	(7.1)	(6.2–8.2)	(23.0)	(21.5–24.5)
South Carolina	10,340	(5.8)	(5.2–6.4)	(13.0)	(12.0–14.1)	(15.6)	(14.8–16.5)	(4.2)	(3.7–4.7)	(6.9)	(6.3–7.5)	(25.5)	(24.3–26.7)
South Dakota	6,755	(2.9)	(2.3–3.5)	(6.9)	(6.0–7.9)	(11.1)	(10.1–12.1)	(2.3)	(1.8–2.9)	(4.3)	(3.5–5.1)	(17.9)	(16.6–19.4)
Tennessee	5,454	(7.5)	(6.5–8.5)	(16.1)	(14.7–17.6)	(19.9)	(18.5–21.4)	(5.7)	(4.9–6.7)	(9.9)	(8.8–11.1)	(31.4)	(29.6–33.2)
Texas	10,468	(5.0)	(4.4–5.7)	(9.5)	(8.6–10.3)	(13.1)	(12.2–14.0)	(3.7)	(3.2–4.2)	(6.2)	(5.6–6.9)	(21.9)	(20.8–23.1)
Utah	12,328	(3.0)	(2.7–3.4)	(9.6)	(9.0–10.3)	(10.0)	(9.4–10.6)	(2.3)	(2.0–2.7)	(4.2)	(3.8–4.6)	(18.9)	(18.0–19.7)
Vermont	6,186	(2.8)	(2.3–3.4)	(9.1)	(8.1–10.3)	(9.0)	(8.2–9.7)	(2.7)	(2.2–3.2)	(5.1)	(4.4–5.9)	(17.8)	(16.6–19.2)
Virginia	8,023	(4.4)	(3.8–5.0)	(8.9)	(8.0–9.8)	(11.7)	(10.9–12.6)	(3.1)	(2.7–3.6)	(5.6)	(5.0–6.2)	(19.6)	(18.5–20.7)
Washington	10,874	(4.0)	(3.6–4.6)	(11.2)	(10.4–12.1)	(12.3)	(11.5–13.1)	(3.7)	(3.2–4.2)	(6.2)	(5.6–6.8)	(22.1)	(21.1–23.2)
West Virginia	5,814	(6.3)	(5.6–7.0)	(15.0)	(13.8–16.2)	(18.8)	(17.8–19.9)	(5.0)	(4.4–5.6)	(10.2)	(9.3–11.2)	(29.8)	(28.4–31.3)
Wisconsin	6,207	(3.0)	(2.4–3.8)	(10.0)	(8.7–11.4)	(11.2)	(10.0–12.5)	(3.1)	(2.5–3.9)	(5.9)	(5.0–7.0)	(19.9)	(18.3–21.6)
Wyoming	6,232	(3.6)	(3.1–4.3)	(8.4)	(7.4–9.5)	(12.3)	(11.2–13.4)	(2.9)	(2.3–3.6)	(5.2)	(4.5–6.0)	(19.5)	(18.1–20.9)

**Abbreviation:** CI = confidence interval.

* Weighted estimates, age-adjusted to the 2000 U.S. standard population.

† Respondents were asked, “Are you blind or do you have serious difficulty seeing, even when wearing glasses?” (vision disability); “Because of a physical, mental, or emotional condition, do you have serious difficulty concentrating, remembering, or making decisions?” (cognition disability); “Do you have serious difficulty walking or climbing stairs?” (mobility disability); “Do you have difficulty dressing or bathing?” (self-care disability); and “Because of a physical, mental, or emotional condition, do you have difficulty doing errands alone such as visiting a doctor’s office or shopping?” (independent living disability). Respondents who refused to answer, reported “don’t know,” and other missing responses were excluded from the analyses.

§ Including the District of Columbia.

¶ Each disability type might not be independent; one respondent might have two or more disability types.

** Respondents with missing information on disability are not included; number of respondents in each demographic group in Table 2 might not add to this number.

**TABLE 2:** Prevalence* of any disability and disability type† by select sociodemographic characteristics among adults aged ≥18 years — Behavioral Risk Factor Surveillance System, United States, 2013

Characteristic	Respondents§ (No.)	Type of disability¶											

		Vision		Cognition		Mobility		Self-care		Independent living		Any	
					
		(%)	(95% CI)	(%)	(95% CI)	(%)	(95% CI)	(%)	(95% CI)	(%)	(95% CI)	(%)	(95% CI)
**Sex**													
Male	190,711	(4.2)	(4.0–4.4)	(9.3)	(9.1–9.6)	(11.3)	(11.0–11.5)	(3.5)	(3.3–3.7)**	(5.0)	(4.8–5.2)	(19.8)	(19.4–20.1)
Female	274,342	(5.0)	(4.9–5.2)	(11.9)	(11.6–12.2)	(14.6)	(14.3–14.9)	(3.7)	(3.5–3.8)	(7.9)	(7.7–8.1)	(24.4)	(24.1–24.8)
**Age group (yrs)** ††													
18–44	129,528	(2.9)	(2.7–3.1)	(10.1)	(9.8–10.4)	(5.5)	(5.2–5.7)	(1.9)	(1.8–2.0)	(4.4)	(4.2–4.6)	(15.7)	(15.3–16.0)
45–64	181,941	(6.5)	(6.2–6.7)	(12.0)	(11.7–12.3)	(18.2)	(17.8–18.5)	(5.6)	(5.4–5.8)	(8.4)	(8.1–8.7)	(26.2)	(25.8–26.6)
≥65	153,584	(6.6)	(6.3–6.9)	(9.9)	(9.5–10.2)	(27.4)	(27.0–27.9)	(5.3)	(5.1–5.5)	(9.8)	(9.5–10.1)	(35.5)	(35.0–36.0)
**Race/Ethnicity**													
White/Non-Hispanic	363,854	(3.6)	(3.5–3.7)	(10.1)	(9.9–10.3)	(12.0)	(11.8–12.2)	(3.1)	(3.0–3.2)	(6.1)	(5.9–6.2)	(20.6)	(20.3–20.9)
Black/Non-Hispanic	37,105	(7.4)	(6.9–7.9)	(13.3)	(12.7–14.0)	(18.7)	(18.0–19.4)	(5.7)	(5.3–6.1)	(9.2)	(8.7–9.7)	(29.0)	(28.1–29.9)
Hispanic	29,371	(7.4)	(6.8–8.0)	(12.1)	(11.5–12.8)	(14.6)	(13.8–15.3)	(4.7)	(4.2–5.1)	(7.3)	(6.8–7.9)	(25.9)	(25.0–26.8)
Other/Non-Hispanic	27,632	(5.5)	(4.8–6.3)	(10.2)	(9.4–11.1)	(11.8)	(10.9–12.7)	(3.4)	(3.0–3.9)	(6.4)	(5.7–7.1)	(21.1)	(20.0–22.3)
**Veteran status**													
Veteran	58,713	(3.9)	(3.5–4.2)	(10.8)	(10.2–11.5)**	(13.4)	(12.8–14.0)**	(3.8)	(3.4–4.2)**	(5.9)	(5.4–6.4)	(22.1)	(21.3–22.9)
Non-veteran	406,010	(4.7)	(4.6–4.9)	(10.6)	(10.4–10.8)	(13.1)	(12.9–13.3)	(3.6)	(3.5–3.7)	(6.7)	(6.5–6.8)	(22.3)	(22.0–22.5)
**Annual household income**													
<$15,000	47,828	(12.4)	(11.8–13.0)	(26.1)	(25.3–27.0)	(29.2)	(28.5–30.0)	(10.1)	(9.6–10.6)	(18.1)	(17.4–18.8)	(46.9)	(46.0–47.9)
$15,000–<$25,000	72,390	(7.3)	(6.9–7.8)	(16.0)	(15.5–16.6)	(20.1)	(19.5–20.6)	(5.7)	(5.4–6.0)	(9.9)	(9.5–10.4)	(33.0)	(32.3–33.7)
$25,000–<$35,000	46,740	(4.6)	(4.2–5.1)	(10.2)	(9.6–10.8)	(14.1)	(13.5–14.7)	(3.6)	(3.2–4.0)	(6.1)	(5.6–6.5)	(23.6)	(22.8–24.4)
$35,000–<$50,000	59,235	(3.2)	(2.9–3.6)	(7.4)	(7.0–8.0)	(10.1)	(9.7–10.6)	(2.3)	(2.1–2.5)	(4.2)	(3.9–4.6)	(17.7)	(17.0–18.3)
$50,000+	176,210	(1.7)	(1.6–1.9)	(4.3)	(4.1–4.5)	(5.9)	(5.7–6.1)	(1.2)	(1.1–1.3)	(2.2)	(2.0–2.3)	(10.8)	(10.5–11.1)
**Employment status**													
Employed	230,472	(2.5)	(2.3–2.6)	(5.6)	(5.4–5.8)	(6.0)	(5.8–6.2)	(1.0)	(0.9–1.1)	(1.7)	(1.6–1.9)	(12.6)	(12.3–12.9)
Unemployed	24,661	(7.2)	(6.5–8.0)	(18.2)	(17.2–19.2)	(17.2)	(16.3–18.2)	(4.8)	(4.2–5.4)	(9.6)	(8.8–10.5)	(33.5)	(32.3–34.7)
Retired/Student/Homemaker	172,389	(4.0)	(3.7–4.4)	(9.4)	(9.0–9.9)	(11.8)	(11.5–12.2)	(2.4)	(2.3–2.6)	(5.4)	(5.1–5.7)	(21.2)	(20.6–21.8)
Unable to work	35,690	(19.1)	(18.0–20.3)	(48.3)	(46.8–49.7)	(60.8)	(59.4–62.2)	(26.9)	(25.6–28.2)	(45.4)	(44.0–46.8)	(82.6)	(81.3–83.8)
**Education level** §§													
<High school	36,615	(10.8)	(10.1–11.4)	(19.9)	(19.1–20.7)	(25.8)	(24.9–26.6)	(8.0)	(7.5–8.5)	(13.5)	(12.9–14.2)	(39.8)	(38.8–40.8)
High school	125,901	(5.4)	(5.1–5.7)	(12.1)	(11.7–12.5)	(16.3)	(15.9–16.7)	(4.4)	(4.2–4.7)	(7.9)	(7.6–8.2)	(26.0)	(25.5–26.5)
Some college	118,275	(4.0)	(3.8–4.3)	(10.2)	(9.9–10.6)	(14.0)	(13.6–14.4)	(3.8)	(3.6–4.0)	(6.7)	(6.5–7.0)	(22.9)	(22.4–23.3)
College graduate	157,878	(2.0)	(1.9–2.1)	(4.3)	(4.1–4.5)	(7.1)	(6.9–7.3)	(1.6)	(1.5–1.8)	(3.0)	(2.9–3.2)	(11.8)	(11.5–12.1)

**Abbreviation:** CI = confidence interval.

* Weighted estimates, age-adjusted to the 2000 U.S. standard population.

† Respondents were asked, “Are you blind or do you have serious difficulty seeing, even when wearing glasses?” (vision disability); “Because of a physical, mental, or emotional condition, do you have serious difficulty concentrating, remembering, or making decisions?” (cognition disability); “Do you have serious difficulty walking or climbing stairs?” (mobility disability); “Do you have difficulty dressing or bathing?” (self-care disability); and “Because of a physical, mental, or emotional condition, do you have difficulty doing errands alone such as visiting a doctor’s office or shopping?” (independent living disability). Respondents who refused to answer, reported “don’t know,” and other missing responses were excluded from the analyses.

§ Respondents with missing information on disability are not included; all groups might not add to the same total number or the overall number in Table 1.

¶ Each disability type might not be independent; one respondent might have two or more disability types.

** Groups not significantly different with p-value ≥0.05 determined by two-sided chi-square test. All other group comparisons were statistically significantly different with p-values <0.05.

†† Estimates not age-adjusted.

§§ Limited to respondents aged ≥25 years.

## Footnotes

* The BRFSS does not include the recommended question on deafness or serious difficulty hearing.

† Based on section 4302 of the Affordable Care Act, the Department of Health and Human Services issued data collection standard guidance to include a standard set of disability identifiers in all national population health surveys. Available at http://aspe.hhs.gov/datacncl/standards/aca/4302/index.pdf.

§ Response rates for BRFSS are calculated using standards set by the American Association of Public Opinion Research Response Rate Formula #4 (http://www.aapor.org/AAPORKentico/AAPOR_Main/media/publications/Standard-Definitions2015_8theditionwithchanges_April2015_logo.pdf). The response rate is the number of respondents who completed the survey as a proportion of all eligible and likely eligible persons. For detailed information, please see the BRFSS Summary Data Quality Report at http://www.cdc.gov/brfss/annual_data/2013/pdf/2013_DQR.pdf.

¶ The questions for specific disability types are: “Are you blind, or do you have serious difficulty seeing, even when wearing glasses?” (vision); “Because of a physical, mental, or emotional condition, do you have serious difficulty concentrating, remembering, or making decisions?” (cognition); “Do you have serious difficulty walking or climbing stairs?” (mobility); “Do you have difficulty dressing or bathing?” (self-care); and “Because of a physical, mental, or emotional condition, do you have difficulty doing errands alone such as visiting a doctor’s office or shopping?” (independent living).

** Two questions from the 1998 BRFSS were used: “Are you limited in any way in any activities because of an impairment or health problem?” and “If you use special equipment or help from others to get around, what type do you use?”

†† The two questions included in the core BRFSS since 2003 are “Are you limited in any way in any activities because of physical, mental, or emotional problems?” and “Do you now have any health problem that requires you to use special equipment, such as a cane, a wheelchair, a special bed, or a special telephone?”

§§ Section 4302 of the Affordable Care Act states “…any federally conducted or supported health care or public health program, activity or survey (including Current Population Surveys and American Community Surveys conducted by the Bureau of Labor Statistics and the Bureau of the Census) collects and reports, to the extent practicable— ‘(A) data on race, ethnicity, sex, primary language, and disability status for applicants, recipients, or participants.’”

¶¶ Disability and Health Data System (http://dhds.cdc.gov/), an online, interactive data tool developed and maintained by CDC on the health of adults with disabilities, will incorporate state-level health data by disability type in an upcoming update of the site.
